# Age Inclusivity: Perceptions by Students, Faculty, and Staff of the Neglected DEI Category

**DOI:** 10.1093/geroni/igaf122.690

**Published:** 2025-12-31

**Authors:** Joann Montepare, Lauren Bowen, Susan Whitbourne, Nina Silverstein

**Affiliations:** Lasell University, Bedford, Massachusetts, United States; University of Massachusetts Boston, Boston, Massachusetts, United States; University of Massachusetts Boston, Boston, Massachusetts, United States; University of Massachusetts Boston, Boston, Massachusetts, United States

## Abstract

Diversity, equity, and inclusion (DEI) efforts in higher education seldom incorporate age-inclusive values, practices, and policies in intentional ways into ongoing programs. However, the need to advance age inclusivity across our colleges and universities has become a pressing issue for many reasons which call for extending attention to this neglected category. The results of a study are described in which a qualitative analysis was conducted of responses by 412 students, faculty, and staff to an open-ended question about age inclusivity. Although some responses indicated that age inclusivity was an unfamiliar concept (12%), many other responses indicated that respondents perceived age inclusivity in higher education as a valuable focus (40%). Differing views included that age inclusivity should not be a focus in higher education for various reasons including that the needs of younger students are more central to the mission of higher education or that the efforts should be “age-blind” just as other DEI efforts should be “color-blind” (26%), or that it can only work if it is enacted appropriately and thoughtfully (12%). Other comments revealed the existence of ageism on campuses, and the need to rethink how higher education refers to age-diverse students and uses language to communicate information about aging to avoid reinforcing ageist thinking. Study results offer several insights about integrating a more age-inclusive focus into broader DEI efforts including the need to raise awareness, make a clear case for the benefits, and utilize meaningful strategies with specific goals.

